# Developing and testing a measure of consultation-based reassurance for people with low back pain in primary care: a cross-sectional study

**DOI:** 10.1186/s12891-016-1144-2

**Published:** 2016-07-12

**Authors:** Nicola Holt, Tamar Pincus

**Affiliations:** Royal Holloway, University of London, Egham Hill, Egham, TW20 0EX UK

## Abstract

**Background:**

Reassurance from physicians is commonly recommended in guidelines for the management of low back pain (LBP), but the process of reassurance and its impact on patients is poorly researched.

We aimed to develop a valid and reliable measure of the process of reassurance during LBP consultations.

**Methods:**

Items representing the data-gathering stage of the consultation and affective and cognitive reassurance were generated from literature on physician-patient communication and piloted with expert researchers and physicians, a Patient and Public Involvement group, and LBP patients to form a questionnaire. Patients presenting for LBP at 43 General Practice surgeries were sent the questionnaire. The questionnaire was analysed with Rasch modelling, using two samples from the same population of recent LBP consultations: the first (*n* = 157, follow-up *n* = 84) for exploratory analysis and the second (*n* = 162, follow-up *n* = 74) for confirmatory testing. Responses to the questionnaire were compared with responses to satisfaction and enablement scales to assess the external validity of the items, and participants completed the questionnaire again one-week later to assess test-retest reliability.

**Results:**

The questionnaire was separated into four subscales: data-gathering, relationship-building, generic reassurance, and cognitive reassurance, each containing three items. All subscales showed good validity within the Rasch models, and good reliability based on person- and item-separations and test-retest reliability. All four subscales were significantly positively correlated with satisfaction and enablement for both samples. The final version of the questionnaire is presented here.

**Conclusions:**

Overall, the measure has demonstrated a good level of validity and generally acceptable reliability. This is the first measure to focus specifically on reassurance for LBP in primary care settings, and will enable researchers to further understanding of what is reassuring within the context of low back pain consultations, and how outcomes are affected by different types of reassurance. Additionally, the measure may provide a useful training and audit tool for physicians. The new measure requires testing in prospective cohorts, and would benefit from further validation against ethnographic observation of consultations in real time.

## Background

Delivering effective reassurance to people presenting with musculoskeletal, or non-specific low back pain (LBP) is recommended by most guidelines, to convey the message that LBP has a good prognosis, there is no need for x-rays, there is no underlying serious pathology, and patients should stay active [1]. These messages are considered to enhance patients’ ability to self-manage and reduce long term disability. Evidence on effective reassurance in LBP remains scarce. A systematic review [2] of prospective cohorts in primary care that measured practitioners’ behaviours during the consultation and their association with patient outcomes found only one study in LBP [3]. The majority of studies included mixed groups of consecutive consultations. The findings from the review suggest that while cognitive reassurance (explaining the aetiology and prognosis and discussing interventions) is associated with better outcomes in primary care, affective reassurance (rapport building, indications of empathy and generic reassuring statements) might improve patient satisfaction, but might result in higher symptom burden later on for patients with non-specific conditions. The authors refer to earlier theoretical work [4] that argues that affective reassurance results in immediate reduction of anxiety, but this in turn leads to reduction in patients’ engagement with cognitive reassurance, breeds dependence on the practitioner, and ultimately results in worse outcomes in the long run. As a result, reassurance of any kind may be expected to increase patients’ immediate satisfaction and enablement, as they leave the consultation still experiencing the beneficial effects of the practitioner telling them that they are going to be fine, but if effective cognitive reassurance has not been properly engaged with, anxiety will recur in the face of ongoing symptoms. Findings from Interviews with low back pain patients [5] supported these conclusions, as they describe patients’ perceptions that only explicit reassurance through explanations about their problem reduced participants’ concerns. The participants in this sample noticed, appreciated, and remembered affective behaviours and wanted to feel that their physician understood them and was taking them seriously, but valued information which would help them to manage their problem more highly.

The impact of physicians’ consultation-based reassurance in LBP warrants further investigation. Even in groups conceptualised as low-risk of long-term pain (those who do not exhibit psychological obstacles to recovery) interventions are not optimal. For example, evidence from a large randomised controlled trial that screened patients for risk, and offered those at low-risk minimal intervention [6], based mainly on education shows that at 4 months 27 % had not recovered, and 37 % had not recovered at 12 months. These findings suggest that for this group interventions can be improved, but this requires better understanding of patients’ needs, and better evidence to develop more effective minimal interventions.

In order to study how consultation-based reassurance impacts on outcomes in LBP, ultimately leading to improved consultations, there is a need to develop a measure of the process. Any measure must be tested in relevant populations (in this case LBP patients) and demonstrate good levels of reliability and validity, in order to be considered an acceptable tool for capturing reassurance. There are a number of instruments designed to measure the content of consultations in primary care, but none focused on reassurance, or on LBP. The aims of this study were:

1. To develop and test a theory-driven reliable and valid questionnaire to assess consultation-related reassurance in LBP, and
2. The subsequent selection of a short version by removing similar items to ensure our final instrument is easily usable.

## Methods

### Generation of items

For the purposes of this review Linton et al.’s [7] definition of reassurance was used:

“reassurance ‘…removes the fears or doubts of (pain/illness); to comfort’. Reassurance always takes place within the dynamics of the interaction between the caregiver who has the intention to reduce worry, and the patient who is concerned. Ultimately, reassurance is achieved if the patient changes his/her behavior, understanding or thoughts.” ([7], pp. 5)

**Fig. 1:**
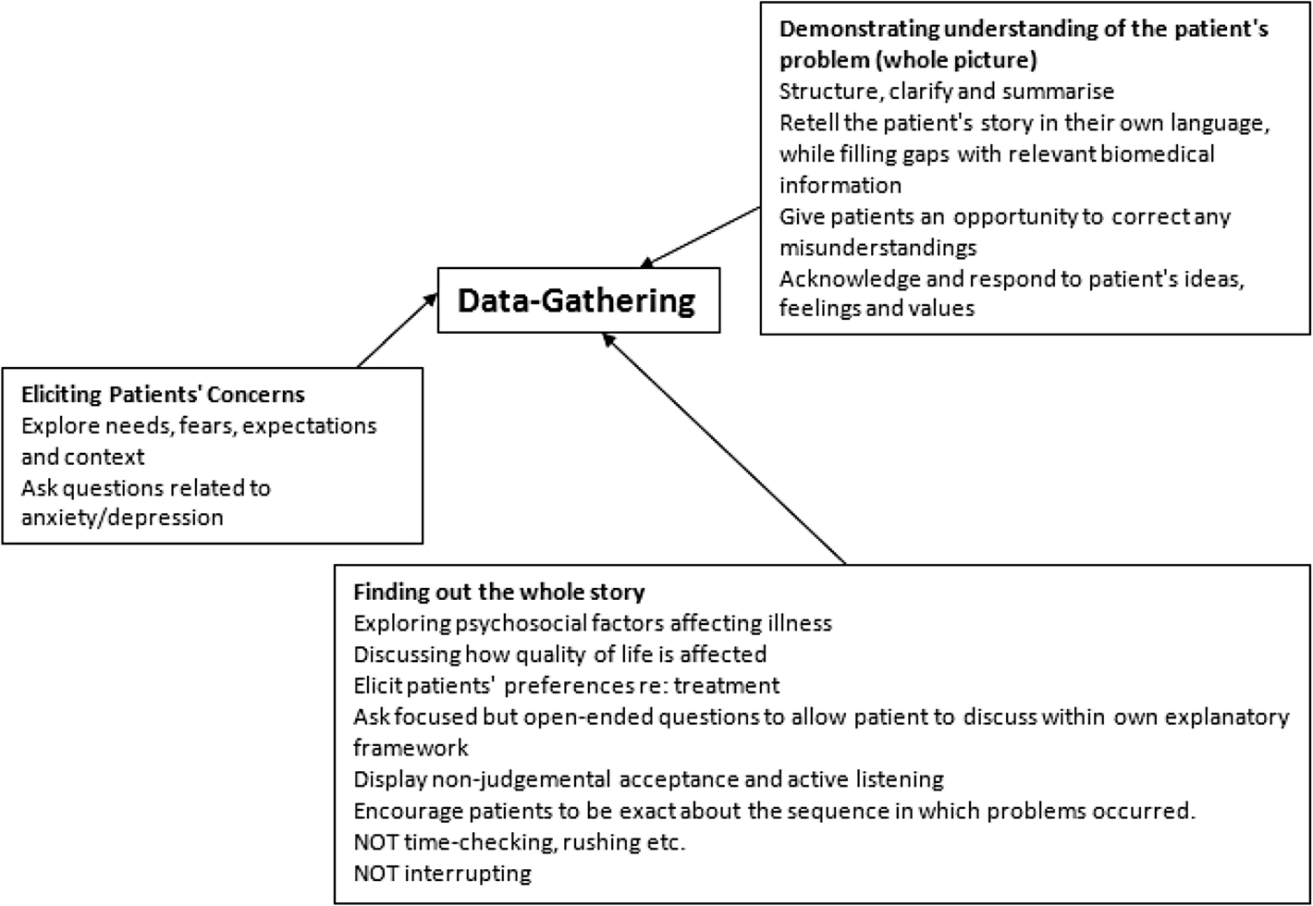
Conceptual map of data gathering

**Fig. 2:**
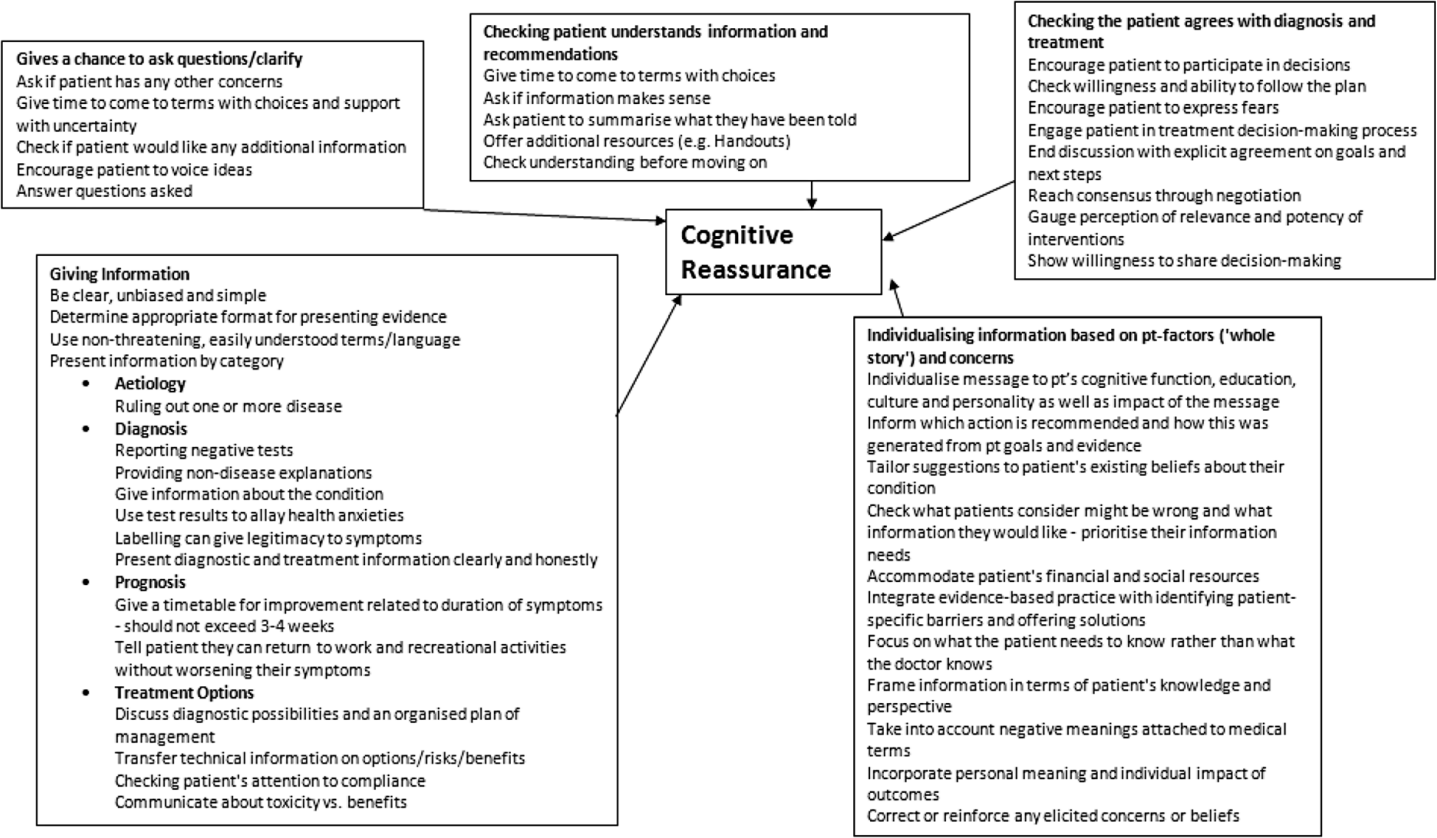
Conceptual map of Cognitive Reassurance

**Fig. 3:**
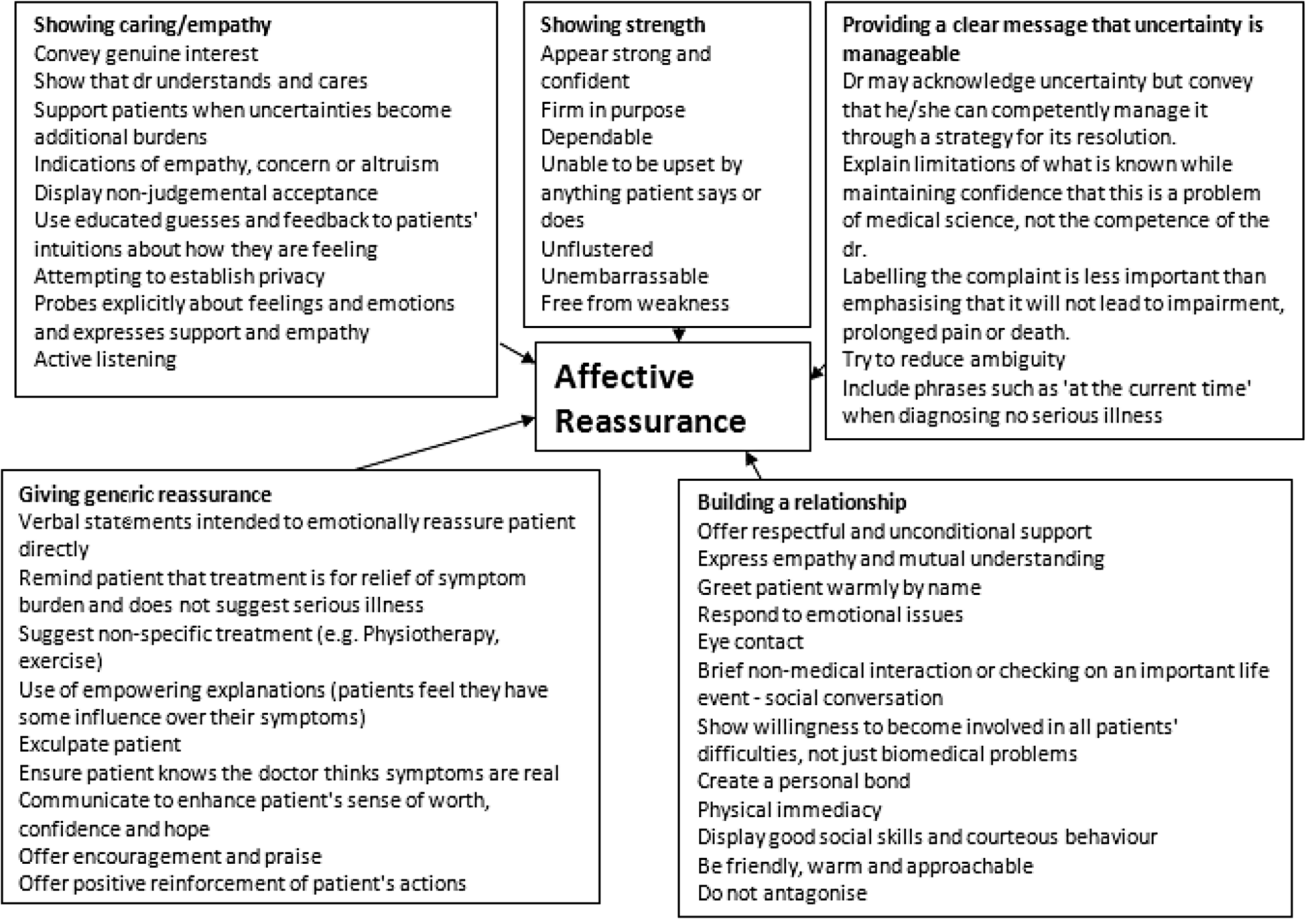
Conceptual map of Affective Reassurance

Therefore, reassurance was defined as any behaviour by a physician which could lead to reduced worry in a concerned patient, and further classified according to the model of reassurance developed by Pincus et al. [2]. In the first instance, specific examples of physicians’ behaviours during consultations were extracted from the literature. We identified theoretical reviews and empirical studies of patient-centred consultation to provide a comprehensive description of the variety of behaviours associated with reassurance. From these reviews, physician behaviours which were theoretically or evidentially associated with improved outcomes post-consultation were extracted. Classification of the identified behaviours according to the model [2] allowed for the formulation of conceptual maps describing different aspects of the consultation. The model describes 3 global concepts: At earlier stages of the consultation, data-gathering included demonstrating understanding of the patient’s problem; eliciting patients’ concerns and finding out the whole story (see Fig. 1). At later stages of the consultation, cognitive reassurance (see Fig. 2) includes giving information about aetiology, prognosis and treatment options; giving patients a chance to ask questions; checking that patients understand the information and the recommendations and matching the information to individual patient concerns and whole story. The final concept (see Fig. 3), Affective Reassurance, includes giving generic reassurance; showing confidence; giving a clear message that uncertainty (in reference to cause/aetiology of the problem, prognosis and/or response to treatment) is manageable; showing care and empathy and building a relationship with the patient.

From these conceptual maps, items were generated under each of the three headings. The items were sent out to a team of expert low back pain researchers, including a psychologist, an osteopath, and two General Practitioners (GPs) for comments. This feedback was used to modify the item pool, change wording where required and add or remove items as recommended. The final pool of items consisted of 30 items: 7 data-gathering; 9 cognitive reassurance; and 14 affective reassurance The items on data gathering appeared first, followed by the items on cognitive and affective reassurance, which randomised. The questions were preceded by the instructions: ‘To what extent did the physician’, and the response mode was a 7 point Likert scale, with the anchors ranging from ‘not at all’ to ‘a great deal’.

Advice on the questionnaire was sought from a Patient and Public Involvement (PPI) group based in Surrey, UK, who indicated that the items were acceptable and understandable. They recommended minor changes in wording, which were applied to the questionnaire. Participants in another study [5] also agreed to read and comment on the questionnaire. Again, the consensus was positive on the item content and presentation.

### Testing of the new questionnaire

#### Participants

Forty-three general practice surgeries in the UK recruited patients presenting for a new episode of LBP between October 2013 and April 2015. Patients were identified by a database search using a search strategy developed specifically for the study by an independent expert company (Holt et al., 2015). The searches were carried out once a month by each practice. The searches were conducted by a researcher at the practice (such as a designated research nurse), and were checked by GPs to ensure that identified patients were eligible and suitable to participate. The practice then sent out a study pack to eligible patients containing the documents outlined below.

The inclusion and exclusion criteria used to identify eligible patients were as follows:

#### Inclusions

Consultation within the previous month.

New episode of acute LBP (duration <6 weeks; no prior episodes within last 6 months) without radiating leg pain and for whom self-management was indicated (ie those not offered follow-up care).

Adult patients (>18 years).

#### Exclusions

Red flag markers.

Cancer.

Cauda equina and ankylosing spondylitis.

Severe disability or end of life disorder.

Pregnancy.

Cognitive impairment or serious mental health problems, which the GP considers could make patients vulnerable and for whom participation would be detrimental.

Previous spinal surgery.

Currently receiving secondary care (physiotherapy, osteopathy, etc.) for the same problem.

Unable to read and speak English.

Those requiring further investigation.

### Materials and procedures

The Questionnaire packs sent to participants contained: a letter of invitation; a study information sheet; a consent form; a questionnaire; and a form to opt in to complete the reassurance questionnaire a second time, one week later, for the purposes of temporal (test-retest) reliability analysis. The following information was collected at the same time as participants’ initial responses to the questionnaire:

#### Demographic information

- Age
- Gender
- Physician gender
- Type of physician (GP or nurse)
- Marital status
- Education level
- Employment status

#### Pain and function

- Length of current episode of LBP
- Whether or not this is the participant’s first episode of LBP
- Number of previous GP consultations for this episode
- Details of any other physician participants had seen since their consultation
- Pain intensity in the week prior to their consultation, rated on the 11-point Pain Numeric Rating Scale ranging from 0 (no pain) to 10 (worst possible pain) (NRS, [8]).
- Functional status was assessed using the Roland-Morris Disability Questionnaire (RMDQ, [9]) which is a well-validated measure of disability in low back pain populations [10].

#### Consultation outcomes

- To measure satisfaction, the Consultation Satisfaction Questionnaire (CSQ, [11]) was used. The CSQ is a validated 9-item questionnaire in which participants respond to statements about how they felt about the consultation on a five-point scale from ‘strongly agree’ to ‘strongly disagree’.
- Enablement was measured with the Patient Enablement Instrument (PEI, [12]) which has been validated for use in primary care populations [13]. The PEI consists of 6 items, rated on a 3-point scale from either ‘much better’ to ‘same or less’ or ‘much more’ to ‘same or less’.

### Analysis

#### Item-response theory

Item Response Theory (IRT), originally developed in educational settings, has grown in popularity within the psychological and health sciences in recent years for constructing measures (eg [14–16]). IRT is based on item response functions, which are mathematical functions describing the relationship between a person’s probable response to a scale item and where he/she falls on the continuum of the construct being measured by that item [15, 16]. IRT models aim to construct measures which accurately assess latent (unobservable) traits, and it is assumed that a person must have a higher level of the trait to score highly on more difficult items. IRT models were originally developed for dichotomous items, but have been extended to include items with nominal response options, such as Likert scales.

The mathematical models used within IRT are independent of sample data, and so comparison of responses across groups becomes possible [17]. Additionally, each item is scrutinised, to reduce redundancy as well as ensuring that the scale is valid and reliable. One of the most commonly used IRT models is the Rasch Measurement Model [18–20], which is used in this analysis. Rasch analysis allows for validity and reliability testing within the same model, and accounts for missing data by using the expected scores (for a person’s ability on a question’s difficulty level) where no score has been given. In this analysis the one-parameter Rasch rating scale model (RSM) is used, which is an extension of the simple (dichotomous) Rasch model for rating scale observations like the present one. The model allows the item difficulty (in this case the extent to which each behaviour is reported to have been present) to be based on the way in which an appropriate group of subjects (ie the patients) actually responded to that question, and establishes the relative difficulty of each item stem in recording the development of an attitude from the lowest to the highest levels the instrument is able to record, ie from response categories 1 to 7 [21, 22].

**Fig. 4:**
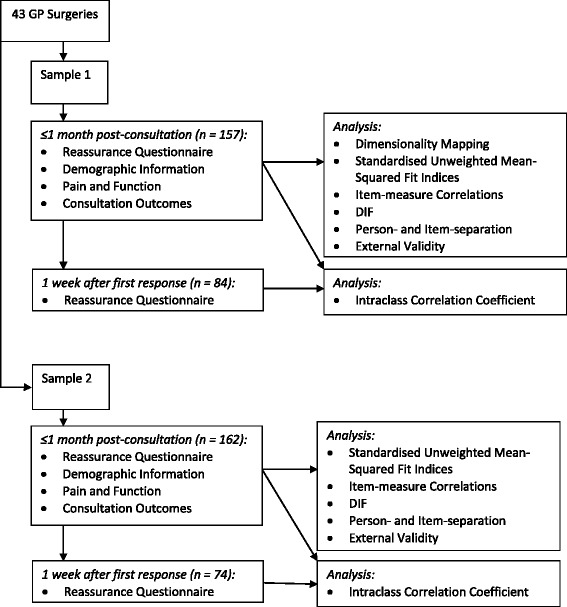
Collection and analysis of data

This study employed a cross-sectional design; all data were taken from participants at a single time-point, with the exception of the reassurance questionnaire which was answered for a second time one week after the first in order to assess test-retest reliability. Two separate samples were obtained for this study: the first 150 participants, referred to as Sample 1, for an exploratory analysis of the questionnaire; the second 150 participants (Sample 2) were new participants recruited from the same pool of practices for confirmatory testing. Potential participants who had already been invited to take part in the study had a study-specific Read code entered into their notes, which allowed us to exclude those already invited from future searches, should they have consulted again within the study period. All analyses were conducted on both samples, with the exception of Dimensionality Mapping (see ‘Structural Validity, below), which identified subscales within the questionnaire from Sample 1’s data only. See Fig. 4 for a representation of the collection and analysis of data for this study. Analyses were conducted using Winsteps version 3.8.1.0 computer software [23] and following the guidance for conducting and reporting Rasch analysis set out by Tennant and Conaghan [24].

#### Validity aspects to be tested

##### Structural validity

testing appraises the fidelity of the scoring structure to the structure of the latent construct domain. Using the first sample, the dimensionality of the questionnaire was measured to ensure that the items were loading onto theoretically meaningful constructs. In line with the first aim of this study (developing and testing a theory-driven reliable and valid questionnaire to assess consultation-related reassurance in LBP) dimensionality Maps were run in Winsteps [23], which assess how much variance is explained by the items as a whole, and provides estimates for clusters which may represent separate dimensions. The Winsteps guide [25] recommends treating item clusters with Eigenvalues of more than 2 as separate subscales, and subsequently running the dimensionality maps again separately for the items which load more than 0.4 on the cluster, and for the remaining items, and so on until no significant clusters remain. The results of each analysis were investigated qualitatively (ie by checking the content of the items) to ensure that item clusters were theoretically meaningful. Any sub-scales identified during this process were adhered to in further analysis, described below.

##### Content validity

refers to the relevance and representativeness of the items of the content upon which they are based. Face validity for items had already been explored through expert review and the use of patient advisory groups. We further tested the content validity of our measure according to the Rasch model using item-measure correlations and standardised unweighted mean-squared fit indices for each subscale separately. Item-measure correlations indicate how well scores on a particular item are consistent with the average score across the remaining items. As advised by Wolfe & Smith [18], correlations of 0.4 and above were considered satisfactory. Standardised unweighted mean-squared fit indices evaluate individual items by comparing their observed and expected values. This tells us how well each item ‘fits’ with the rest of the scale. An Item with a higher score suggests the presence of large residuals in the data, meaning that the item may not be measuring the same construct as the rest of the items. Conversely, items with very low mean-squared fit values indicate the data ‘overfitting’ the model, which could indicate redundancy in our scale. Items with mean-squared fit values exceeding ±2 were examined qualitatively to assess their value to the scale, and removed as indicated, in line with the second aim of the study which was to select a short version of the questionnaire by removing similar items to ensure our final instrument is easily usable.

Differential Item Functioning (DIF) assesses whether items maintain their meaning across different groups of respondents. In other words, whether individuals from different groups respond differently to an item despite having the same ability level. DIF analyses were run across groups according to education level (to ensure that the wording of the question did not discriminate between those of higher and lower educational attainment) and physician gender (to assess whether preconceived expectations of either gender’s behaviour did not influence participants’ responses to the items). Items with DIF *t*-test scores of ±2 or more were to be investigated qualitatively.

##### Reliability

was assessed in two ways, to further address the aim of the study in producing a valid and reliable measure. First, the person- and item-separation and reliability indices built into the Winsteps programme [23] were obtained within the Rasch model. Person separation is used to classify people. Low person separation with a relevant person sample implies that the instrument may not be sensitive enough to distinguish between high and low performers, and more items may be needed. Item separation is used to verify the item hierarchy. Low item separation implies that the person sample is not large enough to confirm the item difficulty hierarchy of the instrument. Winsteps advises that a reliability coefficient of 0.5 is the minimum meaningful reliability, and 0.8 is the minimum required for ‘serious decision-making’. Therefore, subscales with a person- or item-reliability score higher than 0.5 will be considered to show acceptable reliability, and subscales with a person-or item-reliability score higher than 0.8 will be considered to show good reliability.

Secondly, correlational analysis comparing participants’ scores at two time points (post-consultation and one-week later) assessed the temporal reliability of the scale. The interval between responses is important, because too short a gap can result in participants recalling and replicating their responses, and too large a gap may result in recording real changes in patients’ perceptions, understanding and recall. We opted for a time interval of one week between receiving the responses to the questionnaire and sending out the questionnaire again. An intraclass correlation coefficient (ICC) is the most appropriate statistical method for continuous scores. Terwee et al. [26] recommend ICC agreement over ICC consistency because ICC agreement takes systematic error into account. This requires at least 50 participants to provide two sets of responses to the scale [26]. This analysis was conducted in SPSS version 21 [27], and coefficients of 0.7 or higher were considered acceptable [28].

##### External validity

is the degree to which measures are related to external measures of the same, similar, or other constructs. Spearman’s Rho correlations were used to compare our scale with the Consultation Satisfaction Questionnaire (CSQ, [11]) and the Patient Enablement Instrument (PEI, [12, 13]). It was anticipated that the affective reassurance subscale would produce a positive correlation of >0.4 with patient satisfaction, as measured by the CSQ. The cognitive reassurance subscale was expected to produce a positive correlation of >0.4 with patient enablement, as measured by the PEI. These predictions were derived from the theory upon which this questionnaire is based [2, 4], and measuring these correlations further met the first aim of the study, to ensure that the questionnaire was valid, reliable, and fit with current theory.

## Results

### Participants

One hundred and fifty-seven participants returned questionnaires for the first sample; 162 patients provided data for sample 2. Patient characteristics are presented in Table 1.

**Table 1:** Participant Characteristics

	Sample 1	Sample 2
Average age	56.63 (SD 16.64)	53.52 (SD 16.08)
Gender	63.9 % female	63.4 % female
	36.1 % male	36.6 % male
Length of current episode	33.8 % <1 month	24.1 % <1 month
	23.0 % 1–3 months	27.2 % 1–3 months
	11.5 % 4–6 months	11.4 % 4–6 months
	14.2 % 7 months – 3 years	23.4 % 7 months – 3 years
	17.6 % >3 years	13.9 % >3 years
Number of consultations for this episode	47.9 % none	54.4 % none
	31.9 % 1–2	30.9 % 1–2
	14.3 % 3–10	12.5 % 3–10
	5.9 % >10	2.2 % >10
Work status	53.9 % employed (full or part time)	56.2 % employed (full or part time)
	35.7 % retired	32.1 % retired
	3.9 % looking after home/family	3.1 % looking after home/family
	1.9 % unemployed (health reasons)	3.7 % unemployed (health reasons)
	2.6 % unemployed (other)	1.9 % unemployed (other)
	1.9 % student	3.1 % student
Education level	49.0 % obtained higher education degree/certification	44.0 % obtained higher education degree/certification
	18.1 % obtained A levels or equivalent	20.7 % obtained A levels or equivalent
	32.9 % left school at or before 16	35.3 % left school at or before 16
Marital status	65.8 % married/civil partnership	57.8 % married/civil partnership
	7.7 % cohabiting	9.9 % cohabiting
	7.7 % single	14.9 % single
	9.7 % divorced	12.4 % divorced
	6.5 % widowed	5.0 % widowed
	2.6 % other	
Physician type	99.3 % GP	96.3 % GP
	0.7 % nurse practitioner	3.8 % nurse practitioner
Physician gender	52.9 % male	50.9 % male
	47.1 % female	49.1 % female
First episode?	26.1 % yes	27.2 % yes
	73.9 % no	72.8 % no
Average pain intensity in the last week (/10)	7.14 (SD 2.02)	7.06 (SD 2.06)
RMDQ score (/24)	10.34 (SD 5.73)	10.10 (SD 5.98)

### Structural validity: dimensionality analyses

**Fig. 5:**
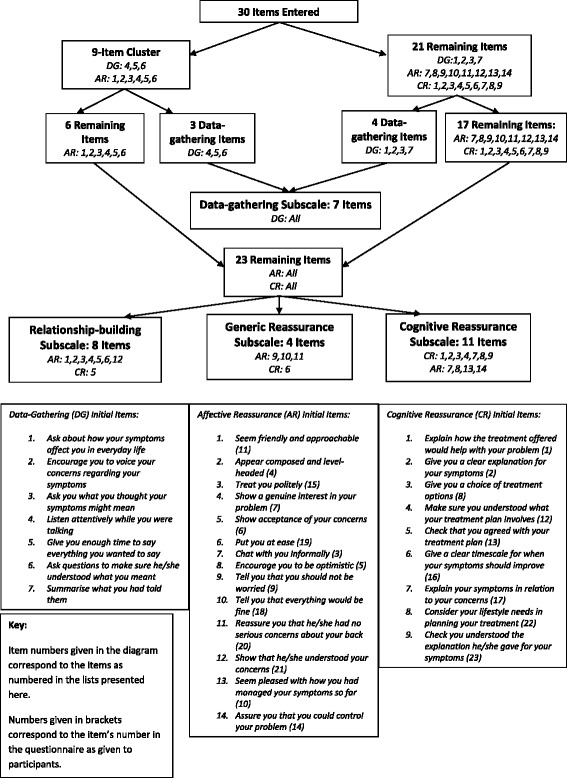
Dimensionality Mapping results

A dimensionality map of the responses of Sample 1 on the entire scale revealed that it was not unidimensional. See Fig. 5 for a representation of the identified dimensions within the questionnaire.

1. First, a major cluster was identified consisting of 9 items. A second dimensionality map of this cluster showed that these items were also multidimensional, and separated them into two clusters, one consisting of 3 data-gathering items and the other of 6 affective reassurance items.
2. A dimensionality map of the remaining 21 items separated the other 4 data-gathering items from the rest of the scale. *As depicted in* Fig. 5*, the dimensionality analyses separated the data-gathering items from the remainder of the item pool at the second stage. The three items in the first cluster were:*
  4. *Listen attentively while you were talking*
  5. *Give you enough time to say everything you wanted to say*
  6. *Ask questions to make sure he/she understood what you meant*
  The four items from the remaining pool were:
  1. *Ask about how your symptoms affect you in everyday life*
  2. *Encourage you to voice your concerns regarding your symptoms*
  3. *Ask you what you thought your symptoms might mean*
  4. *Summarise what you had told them*
  *As the key concepts underpinning data-gathering (demonstrating understanding of the patient’s problem; eliciting patients’ concerns and finding out the whole story) were represented across both of these clusters, they were assessed as not being qualitatively different enough to warrant two subscales. Because the dimensionality analyses had separated the data-gathering items from the items which concerned the later stages of the consultation, the researchers made the decision to place all the items together in subsequent analyses, with the understanding that analysis of fit indices would identify any items which did not fit with the overall subscale.*
3. Next, dimensionality maps were run on the 23 data-giving items from the scale, and provided three clusters. Out of 30 items, 24 mapped onto constructs hypothesised in the model (highlighted in bold in Table 2). All of the items were retained at this stage for further analysis. The items included in each newly identified subscale are presented in Table 2.

**Table 2:** All Items entered into Rasch Analyses

Cluster 1 (Data-Gathering)	Cluster 2 (Relationship-Building)	Cluster 3 (Generic Reassurance)	Cluster 4 (Cognitive Reassurance)
**1. Ask about how your symptoms affect you in your everyday life ** *4.10 (1.92)*	**4. Appear composed and level-headed ** *6.06 (1.04)*	**9. Tell you that you should not be worried ** *3.96 (2.05)*	**1. Explain how the treatment offered would help with your problem ** *4.51 (1.78)*
**2. Encourage you to voice your concerns regarding your symptoms ** *4.50 (1.82)*	**11. Seem friendly and approachable ** *5.82 (1.31)*	16. Give a clear timescale for when your symptoms should improve *3.88 (2.15)*	**2. Give you a clear explanation for your symptoms ** *4.36 (1.88)*
**3. Ask you what you thought your symptoms might mean ** *3.54 (1.97)*	**7. Show a genuine interest in your problem ** *5.38 (1.61)*	**18. Tell you that everything would be fine ** *3.52 (2.09)*	3. Chat with you informally *4.89 (4.47)*
**4. Listen attentively while you were talking ** *5.75 (1.27)*	**15. Treat you politely ** *6.24 (1.01)*	**20. Reassure you that he/she had no serious concerns about your back ** *4.38 (2.02)*	5. Encourage you to be optimistic *4.75 (1.71)*
**5. Give you enough time to say everything you wanted to say ** *5.56 (1.50)*	**6. Show acceptance of your concerns ** *5.30 (1.56)*		**8. Give you a choice of treatment options ** *3.72 (2.12)*
**6. Ask questions to make sure he/she understood what you meant ** *5.18 (1.72)*	**19. Put you at ease ** *5.13 (1.79)*		10. Seem pleased with how you had managed your symptoms so far *4.26 (1.89)*
**7. Summarise what you had told them ** *4.77 (1.86)*	13. Check that you agreed with the treatment plan *4.85 (1.97)*		**12. Make sure you understood what your treatment plan involves ** *4.95 (1.94)*
	**21. Show that he/she understood your concerns ** *5.12 (1.80)*		14. Assure you that you could control your problem *4.22 (2.01)*
			**17. Explain your symptoms in relation to your concerns ** *4.40 (2.04)*
			**22. Consider your lifestyle and needs in planning your treatment ** *4.18 (2.13)*
			**23. Check you understood the explanation he/she gave for your symptoms ** *4.65 (1.96)*

Items highlighted in bold are those which mapped directly to the theoretical constructs in the model

Numbers given in italics: *mean (SD)*

### Content validity and reliability

Assessment using the principles of Rasch measurement was conducted on each subscale.

#### Data-gathering

Seven items were entered into the Standardised unweighted mean-squared fit indices analysis and calculation were carried omitting problematic items until both infit and outfit for the remaining items fell within acceptable ranges. The final model, which included items 2, 4 and 7 (encourage you to voice your concerns regarding your symptoms; listen attentively while you were talking; and summarise what you had told them), showed good fit for all items and was used in the remainder of analyses. Item-measure correlations were calculated for the reduced subscale, and were found to be strong: 0.88, 0.80, and 0.88 for items 2, 4 and 7 respectively. This was then repeated in the second sample, confirming the fit with all standardised unweighted mean-squared fit indices under the ±2 threshold for problematic items, and item measure correlations ranging between 0.82–0.92.

DIF statistics were calculated for items 2, 4 and 7 to assess whether different items were answered differently by participants from different groups. For both samples, tests for education level and physician level were non-significant.

Reliability was assessed for this subscale using Rasch person- and item-separation statistics and ICCs comparing scores on the items one week after one another. For sample 1, the person separation was 2.08 (reliability coefficient 0.81), and the item separation was 8.67 (reliability coefficient 0.99), indicating a good level of reliability. Reliability remained high for sample 2: person separation 2.26 (reliability coefficient 0.8); item separation 8.65 (reliability coefficient 0.99). The results for Average Measures ICC with two-way mixed agreement are presented in Table 3. Correlations were all above the acceptable level of 0.70, and so the subscale can be considered to have good test-retest reliability.

**Table 3:** Intraclass Correlation Coefficients (ICCs) for all subscales

	ICC Sample 1	ICC Sample 2
*Data gathering*		
Item 2	0.85, *n* = 75 (74,74)	0.82, *n* = 68 (67,67)
Item 4	0.83, *n* = 74 (73,73)	0.70, *n* = 67 (66,66)
Item 7	0.77, *n* = 74 (73,73)	0.75, *n* = 68 (67,67)
Whole subscale	0.90, *n* = 76 (75,75)	0.81, *n* = 68 (67,67)
*Relationship building (Subscale 1)*		
Item 7	0.87, *n* = 155 (154,154)	
Item 19	0.84, *n* = 155 (154,154)	
Item 21	0.88, *n* = 154 (153,153)	
Whole subscale	0.93, *n* = 153 (152,152)	
Relationship-building (Subscale 2)		
Item 4	0.78, *n* = 156 (155,155)	
Item 6	0.80, *n* = 156 (155,155)	
Item 15	0.86, *n* = 156 (155,155)	
Whole subscale	0.88, *n* = 156 (155,155)	
*Generic reassurance*		
Item 9	0.87, *n* = 71 (70,70)	0.82, *n* = 68 (67,67)
Item 18	0.90, *n* = 68 (67,67)	0.83, *n* = 66 (65,65)
Item 20	0.89, *n* = 73 (72,72)	0.77, *n* = 68 (67,67)
Whole subscale	0.91, *n* = 73 (72,72)	0.87, *n* = 68 (67,67)
*Cognitive reassurance*		
Item 1	0.82, *n* = 72 (71,71)	0.82, *n* = 65 (64,64)
Item 12	0.82, *n* = 71 (70,70)	0.79, *n* = 65 (64,64)
Item 23	0.85, *n* = 72 (71,71)	0.79, *n* = 66 (65,65)
Whole subscale	0.82, *n* = 73 (72,72)	0.88, *n* = 66 (65,65)

#### Relationship building

Eight items were entered and the procedure described repeated. The final model, made up of items 7, 19 and 21 (show a genuine interest in your problem; put you at ease; and show that he/she understood your concerns respectively), showed good fit for all items and was used in analysis of sample 2. Item-measure correlations were calculated for the reduced subscale, and were found to be 0.86, 0.91 and 0.91 for items 7, 19 and 21 respectively, suggesting that each of the items correlated strongly with the final, reduced subscale. For sample 2, items 7 and 19 showed standardised mean-squared fit indices outside of the acceptable ranges of ±2, suggesting the presence of large residuals within the data. As removal of either of these items would leave only two in the subscale, it was decided instead that all of the original Relationship-building items (see previous page) would be re-entered using sample 2’s data, to assess whether a different combination of the items might better represent the construct. This model would then be re-checked using the data from sample 1. The item-measure correlations for a subscale containing items 4, 11, 15 and 6 were 0.87, 0.88, 0.82, and 0.90 respectively. When these items were entered into Winsteps using sample 1’s data, item 11 was misfitting (infit −2.3; outfit −2.4). This was removed, and the remaining three items showed good fit for both samples. The three items in the second reduced subscale (appear composed and level-headed; treat you politely; and show acceptance of your concerns) Therefore, both subscales were analysed using the combined data from Sample 1 and 2 before a decision was reached on which to include in the final questionnaire. Both subscales showed acceptable fit statistics and strong item-measure correlations.

DIF statistics showed that when separated by education level, or physician gender, variation was evenly spread amongst groups for both subscales, with no significant *t*-test results.

For the first subscale, person- and item-reliability were both above the threshold for good reliability (0.82 and 0.89, respectively). However, for the second subscale person reliability was 0.77, and therefore failed to meet the standard for good reliability of >0.8, although item-separation was good at 0.99. Test-retest reliability was strong for both subscales (see Table 3).

Overall, both potential subscales performed well when analysed using samples 1 and 2 combined. However, the second subscale showed weaker person-separation than the first, which can be indicative of a ceiling effect. As the items in the first subscale were felt to be more qualitatively meaningful in the context of relationship-building, this subscale was included in the final questionnaire.

#### Generic reassurance

Four Items were included in the Standardised unweighted mean-squared fit indices analysis of the generic reassurance subscale. The final model, made up of items 9, 18 and 20 (tell you that you should not be worried; tell you that everything would be fine; and reassure you that he/she had no serious concerns about your back, respectively), showed good fit for all items and was used in subsequent analyses. Item-measure correlations for the reduced subscale were 0.89, 0.90 and 0.85 for items 9, 18 and 20 respectively, suggesting that the items correlated well with overall subscale. The subscale showed good fit when tested again with the data from sample 2. DIF statistics for both samples sample 1 showed that variation was evenly spread amongst groups for education and physician gender.

The generic reassurance subscale showed good reliability. For the first sample, person separation was 2.12 (reliability coefficient 0.82) and the item separation was 4.15 (reliability coefficient 0.95). For the second sample, the person separation was 2.07 (reliability coefficient 0.81) and the item separation was 4.67 (reliability coefficient 0.96). ICC scores are shown in Table 5.15, and demonstrate good test-retest reliability for this subscale (Table 3).

#### Cognitive reassurance

Eleven items were entered into the standardised unweighted mean-squared fit indices analysis. The final model, made up of items 1, 12 and 23 (explain how the treatment offered would help with your problem; make sure you understood what your treatment plan involves; and check you understood the explanation he/she gave for your symptoms, respectively), showed good fit for all items and was used in subsequent analyses. Item-measure correlations were 0.84, 0.81, and 0.84 for items 1, 12 and 23 respectively, suggesting that the items correlated well with the overall subscale. Fit statistics and Item-measure correlations remained at acceptable levels using the data from sample 2. As for the other sub-scales, education level and practitioner gender did not influence responses in either sample.

Person- and item-separation indices were within acceptable ranges for sample 1: the person separation was 2.04 (reliability coefficient 0.81) and the item separation was 2.48 (reliability coefficient 0.86). For sample 2, the person separation was 1.82 (reliability coefficient 0.77) and the item separation was 1.36 (reliability coefficient 0.65). Although the reliability scores for sample 2 fell above the minimum meaningful level of 0.5, they failed to reach to acceptable standard of 0.8. ICCs, however, were all strong for this subscale and indicate acceptable test-retest reliability (table X).

### External validity

All four subscales were significantly positively correlated with satisfaction and enablement, for both samples (Table 4). The hypotheses that affective reassurance (in this case split into relationship-building and generic reassurance) would show a positive correlation >0.4 with satisfaction, and that cognitive reassurance would show a positive correlation >0.4 with enablement were both supported. The final questionnaire is presented in Table 5.

**Table 4:** Correlations between Reassurance Subscales and Satisfaction and Enablement Scales

	Total Satisfaction Score (CSQ)	Total enablement score (PEI)
*Sample 1*		
Data Gathering, *n* = 156	0.71^a^	0.43^a^
Generic Reassurance, *n* = 151	0.54^a^	0.42^a^
Cognitive Reassurance, *n* = 156	0.80^a^	0.48^a^
*Sample 2*		
Data Gathering, *n* = 162	0.77^a^	0.43^a^
Generic Reassurance, *n* = 160	0.45^a^	0.46^a^
Cognitive Reassurance, *n* = 162	0.76^a^	0.52^a^
*Combined Samples*		
Relationship-building Subscale 1, *n* = 312	0.81^a^	0.52^a^

^a^correlation significant at *p* < 0.05

**Table 5:** Final reassurance questionnaire

Data-gathering subscale	Relationship-building subscale	Generic reassurance subscale	Cognitive reassurance subscale
*To what extent did the physician …*			
Encourage you to voice your concerns regarding your symptoms	Show a genuine interest in your problem	Tell you that you should not be worried	Explain how the treatment offered would help with your problem
Listen attentively while you were talking	Put you at ease	Tell you that everything would be fine	Make sure you understood what your treatment plan involves
Summarise what you had told them	Show that he/she understood your concerns	Reassure you that he/she had no serious concerns about your back	Check you understood the explanation he/she gave for your symptoms

## Discussion

The aims of this study were to develop and test a theory-driven reliable and valid questionnaire to assess consultation-related reassurance in LBP. Data reduction, using Rasch analysis resulted in a 12 item questionnaire. Overall, the questionnaire performed well, with good content validity, consistent responses across groups, and acceptable reliability. The final questionnaire represents four distinct aspects of reassurance during consultations: data gathering, relationship building, generic reassurance, and cognitive reassurance.

The four sub-categories map on to the model of reassurance proposed by Pincus et al. (2013). The first two, data gathering and relationship building can be considered to provide implicit reassurance, while the latter can be conceptualised as explicit reassurance. According to Coia and Morley (1998), relationship building and generic reassurance would fall into the category of affective reassurance, combining verbal and non-verbal behaviours. Coia and Morley do not mention data gathering behaviours, possibly because they consider these as attempts to elicit information about the presenting problem, rather than attempts to understand the whole person’s story, including their concerns and the implications on their lives. As such, we consider that the items in the data-gathering sub-scale also represent implicit reassurance, as they convey the patients perception that they have had the opportunity to voice their concerns, and that they have been listened to.

### Strengths and limitations

The split of the four subscales, whilst indeed different from the initial three-construct structure of the overall item pool, we feel is a strength of the tool rather than a weakness. Two of the original subscales were retained: data-gathering and cognitive reassurance; while the items which were at first grouped together under the umbrella term ‘affective reassurance’, to represent all emotionally-based attempts to reduce patients worry, were found to represent two distinct constructs: relationship-building and generic reassurance. Within Coia and Morley’s [4] conceptualisation of reassurance, they describe affective reassurance as a combination of non-verbal cues which are “largely synonymous with the doctor’s manner” and direct verbal statements intended to emotionally reassure. These two aspects of affective reassurance are represented within our final questionnaire structure. Additionally, the separation of relationship-building behaviours from generic reassurance statements maps to the distinction between implicit (unstated but perceived by patients) and explicit (direct and often verbal) reassurance found in earlier qualitative work [5]. Therefore, the final, four-construct questionnaire provides more specificity in evaluating the model than the original structure in which affective reassurance was considered a single construct.

As in all questionnaire development using data reduction techniques, we aim to produce a small set of items that nonetheless captures the most salient items to describe the sub-scales in which they are placed. For this reason our original pool of items includes replication and slightly different voicing of the same item. We aim to exclude most of the items because we want to have a questionnaire that is low burden to patients and therefore usable in research. One of the most pressing problems in the study of psychosocial factors in pain (much like all research in patient groups) is missing data and attrition due to inclusion of too many questionnaires, and questionnaires that are unnecessarily long. The final 12 items included in this questionnaire all showed good fit with the other items in their subscales as measured using standardised unweighted mean-squared indices and item-measure correlations; acceptable reliability; no evidence of differential item functioning, and good external validity when compared with established consultation outcome measures.

Although the sub-scales were shown to have good reliability and validity, we have some concerns about their ability to comprehensively capture all aspects of the consultation. For example, relationship-building was one of the key skills extracted from the literature review, involving emotion-based behaviours such as empathising, being supportive, and forming a bond. The benefits of forming therapeutic relationships with patients are well-reported (eg [29–33]). However, the items produced by our analysis appears more superficial, reflecting the practitioners’ ability to convey confidence, act politely and acknowledge patients’ concerns. Reliability was assessed for all subscales using Rasch estimates of reliability and ICC scores comparing responses to the items given one week apart. While test-retest reliability was demonstrated for all items and subscales, Rasch estimations of reliability were mixed. Specifically, the cognitive reassurance subscale fell just short of the higher standard of reliability (>0.8) when analysed using Sample 2’s data. We acknowledge that this is preliminary work, and that the questionnaire requires further validation to ensure full confidence in its ability to reliably measure the different facets of reassurance.

The study utilised two separate samples for the analysis. While this enabled re-testing findings in a new sample, it could be argued that both samples could be expected to perform similarly, as they were drawn from the same population presenting to the same practices. However, the samples were recruited from 43 general practices, in a large geographical spread and diverse socio-economical catchment populations. This argument is supported by Differential Item Functioning (DIF) analysis, which tests the different probability within groups of endorsing a particular item. All four subscales showed no presence of DIF for either participant education level or physician gender, meaning that responses did not differ significantly across respondents within different groups on these variables. The absence of DIF for participant education is encouraging, as it is essential that a questionnaire is understandable to people from all educational backgrounds [34]. Responses from participants whose physicians had been of different genders were examined as there are documented differences in the ways male and female physicians communicate with patients, with female physicians more likely to engage in empathetic and partnership-building behaviours [35]. Additionally, physician gender has been shown to affect patient satisfaction outside of the effects of patient characteristics and physician behaviours [36], suggesting that patients may hold expectations for physicians of different genders which affect their perceptions of the care they receive. However, all four subscales were resistant to these effects and remained consistent whether the physician in question was male or female.

Scores on all four subscales were correlated with scores on established consultation measures for satisfaction (CSQ, [11]) and enablement (PEI, [12]). All showed significant positive correlations with both instruments for both samples, demonstrating good external validity for the scale. Correlations between the reassurance subscales and enablement were weaker than those between reassurance and satisfaction. Reassurance represents a minimal intervention by GPs, and it may be the case that more intensive intervention is required to enable some patients, particularly those who are considered higher risk for chronicity [6]. Cognitive reassurance was related more strongly than the other subscales to enablement. This finding supported both the hypothesis that the two would be correlated, and the model of reassurance which posits that cognitive reassurance equips patients with the knowledge and skills to manage their problem [2]. Surprisingly, although the generic reassurance subscale was significantly correlated with satisfaction, it showed the weakest correlations of the four subscales in both samples. It was predicted that this type of reassurance would particularly increase satisfaction as it produces immediate reductions in anxiety [4]. The relationship between generic reassurance and satisfaction remains problematic: contradictory evidence was found in a systematic review of prospective cohorts in primary care (Pincus et al., 2013), with three studies showing a positive association between the two, and two studies showing negative associations.

An important limitation of the current study is the delay between consultation and recruitment, due to electronic searches being carried out on a monthly basis. To truly capture participants’ perceptions of reassurance administration of the measure should take place at consultation exit. In addition, participants were included in this sample with both acute and chronic low back pain. A sample of acute cases only (ie people presenting with their first episode of LBP) would be more informative, to avoid contamination from previous consultations.

## Conclusions

Overall, the measure has demonstrated a good level of validity and generally acceptable reliability. This is the first of its kind to focus specifically on reassurance for LBP in primary care settings, and will enable researchers to further their understanding of what is reassuring within the context of low back pain consultations, and how outcomes are affected by different types of reassurance. Additionally, since reassurance is recommended by various guidelines for low back pain (eg [1, 37, 38]) the measure may provide a useful training and audit tool for physicians. The new measure requires testing in prospective cohorts, and would benefit from further validation against ethnographic observation of consultations in real time.

## Abbreviations

GP, General Practitioner; LBP, Low Back Pain; PPI, Patient and Public Involvement
